# Supplementary material for: Proteomics of Salt Gland–Secreted Sap Indicates a Pivotal Role for Vesicle Transport and Energy Metabolism in Plant Salt Secretion
Source: Int J Mol Sci. 2022 Nov 11;23(22):13885. doi: 10.3390/ijms232213885 (PMC9693062; doi:10.3390/ijms232213885)
Supplement: Supplementary file 1 [file ijms-23-13885-s001.zip › ijms-1983618-supplementary.pdf]

Supplementary Table S1. Photosynthetic-related proteins in the secreted sap of *L. bicolor* salt glands treated with 0 and 200 mM NaCl.

Number	Accession	Description	MW (kDa)	Con-trol	NaCl
1	A0A0F7G6K5	Ribulose bisphosphate carboxylase large chain (Fragment) OS=Paronychia herniari-oides	47.9	+	+
2	A0A0H3Y5V0	Ribulose-1,5-bisphosphate carboxylase/oxy-genase large subunit (Fragment) OS=Delo-sperma peglerae	8.7523	+	-
3	A0A0J8B7T8	Glucose-1-phosphate adenylyltransferase OS=Beta vulgaris subsp. vulgaris	56.502	+	-
4	A0A0J8BAT7	Alpha-carbonic anhydrase domain-containing protein OS=Beta vulgaris subsp. vulgaris	31.949	+	-
5	A0A0K9QST5	Malate dehydrogenase OS=Spinacia oleracea	37.821	+	-
6	A0A068ELN9	Ribulose bisphosphate carboxylase large chain (Fragment) OS=Limonium dragon-ericum	50.151	+	+
7	A0A0K9RW58	Chlorophyll a-b binding protein, chloroplastic OS=Spinacia oleracea	30.973	+	-
8	A0A0K9S1L6	Glycosyltransferase OS=Spinacia oleracea	53.801	+	-
9	A0A140ETK2	Ribulose-1,5-bisphosphate carboxylase/oxy-genase large subunit (Fragment) OS=Aerva lanata	17.336	+	-
10	A0A346TN31	Ribulose-1,5-bisphosphate carboxylase/oxy-genase large subunit (Fragment) OS=Atriplex vesicaria	9.2556	+	-
11	A0A411JQT5	Ribulose bisphosphate carboxylase large chain OS=Dioncophyllum thollonii	54.893	+	-
12	A0A411JS61	Cytochrome f OS=Limonium tenellum	35.23	+	-
13	A0A411JYF9	Photosystem II protein D1 OS=Simmondsia chinensis	39.764	+	-
14	A0A411K5C3	Photosystem II D2 protein OS=Rhabdoden-dron amazonicum	39.865	+	-
15	A0A411L863	Cytochrome b6 (Fragment) OS=Lophiocarpus polystachyus	23.58	+	-
16	A0A4D6DHA3	Photosystem I iron-sulfur center OS=Rumex acetosa	9.3818	+	-
17	A0A5C1D6C9	Photosystem I P700 apoprotein A2 OS=Pseu-dostellaria palibiniana	86.338	+	-
18	A0A650AK20	Ribulose bisphosphate carboxylase large chain OS=Limonium aureum	52.67	+	+
19	B2XWP9	Photosystem II CP47 reaction center protein OS=Fagopyrum esculentum subsp. ancestrale	56.123	+	-

20	F2WAU2	Ribulose bisphosphate carboxylase large chain (Fragment) OS=Tecticornia disarticulate	49.537	+	-

The above table continues

21	F6KXD6	Photosystem II CP43 protein (Fragment) OS=Myricaria germanica	16.899	+	-
22	G0WXT9	Ribulose-1,5-bisphosphate carboxylase/oxygenase large subunit (Fragment) OS=Amaranthus deflexus	33.481	+	-
23	H2BLW9	Photosystem I P700 chlorophyll a apoprotein A1 OS=Silene noctiflora	83.14	+	-
24	I3XLX8	Ribulose bisphosphate carboxylase large chain (Fragment)	45.152	+	-
25	I6WY50	Ribulose bisphosphate carboxylase large chain (Fragment) OS=Limonium sinense	46.023	+	-
26	O47047	Ribulose bisphosphate carboxylase large chain (Fragment) OS=Eriogonum flavum	49.73	+	-
27	P00298	Plastocyanin OS=Rumex obtusifolius	10.354	+	-
28	P16016	Carbonic anhydrase, chloroplastic OS=Spinacia oleracea	34.569	+	-
29	A0A3S9XK16	Ribulose bisphosphate carboxylase large chain (Fragment) OS=Plumbago zeylanica	46.635	+	-
30	P28410	Ribulose bisphosphate carboxylase large chain (Fragment) OS=Drosera petiolaris	48.899	+	-
31	P93262	Phosphoglucomutase, cytoplasmic OS=Mesembryanthemum crystallinum	63.445	+	-
32	Q1W6C5	Class Ib chitinase OS=Limonium bicolor	34.13	+	-
33	Q38713	Chlorophyll a-b binding protein, chloroplastic OS=Amaranthus hypochondriacus	28.612	+	-
34	Q6A159	Ribulose bisphosphate carboxylase small chain OS=Limonium gibertii	17.672	+	+
35	Q6A160	Ribulose bisphosphate carboxylase small chain (Fragment) OS=Limonium gibertii	17.627	+	-
36	Q6A161	Ribulose 1,5 bisphosphate carboxylase/oxygenase, large subunit OS=Limonium gibertii	53.413	+	-
37	Q6EYP6	Cytochrome b-559 alpha subunit (Fragment) OS=Spinacia oleracea	8.1121	+	-
38	Q9AVF2	Chlorophyll a-b binding protein, chloroplastic (Fragment) OS=Amaranthus tricolor	16.647	+	-
39	A0A410S7A2	Ribulose-1,5-bisphosphate carboxylase/oxygenase large subunit (Fragment) OS=Achyranthes aspera	12.178	+	-
40	A0A411JS26	ATP synthase CF0 subunit I OS=Limonium tenellum	21.078	+	-

Supplementary Table S2. Respiration-related proteins in the secreted sap of *L. bicolor* salt glands treated with 0 and 200 mM NaCl.

Number	Accession	Description	MW (kDa)	Control	NaCl
1	A0A0K9QFF9	Fructose-bisphosphate aldolase OS=Spinacia oleracea	42.665	+	-
2	A0A0K9QST5	Malate dehydrogenase OS=Spinacia oleracea	37.821	+	-
3	A0A1B2LUN5	Glyceraldehyde-3-phosphate dehydrogenase OS=Haloxylon ammodendron	42.913	+	-
4	A0A411JPW9	ATP synthase subunit beta OS=Ceratostigma willmottianum	53.874	+	+
5	A0A411JPX2	ATP synthase subunit alpha OS=Ceratostigma willmottianum	55.662	+	+
6	A0A411JRY5	ATP synthase subunit alpha OS=Limonium tenellum	55.584	+	-
7	A0A411JS23	ATP synthase subunit beta OS=Limonium tenellum	53.218	+	-
8	D3WEW2	ATP synthase subunit alpha OS=Plumbago auriculata	55.647	+	+
9	F1LK13	Xanthine dehydrogenase (Fragment) OS=Plumbago auriculata	45.51	+	+
10	G4WNY3	NAD(P)H-quinone oxidoreductase subunit 5, chloroplastic (Fragment) OS=Stegnosperma halimifolium	57.819	+	-
11	G5DVX3	Phosphoglycerate kinase (Fragment) OS=Silene latifolia	51.21	+	-
12	G8A3M7	ATP synthase subunit beta (Fragment) OS=Alluaudia procera	50.753	+	-
13	H8Y672	ATP synthase subunit alpha OS=Silenevulgaris	58.181	+	-
14	P12860	Glyceraldehyde-3-phosphate dehydrogenase B, chloroplastic OS=Spinacia oleracea	48.125	+	-
15	P17878	Glyceraldehyde-3-phosphate dehydrogenase, cytosolic OS=Mesembryanthemum crystallinum	36.587	+	-
16	P19866	Glyceraldehyde-3-phosphate dehydrogenase A, chloroplastic OS=Spinacia oleracea	43.023	+	-
17	P27774	Phosphoribulokinase, chloroplastic OS=Mesembryanthemum crystallinum	44.114	+	-
18	P29409	Phosphoglycerate kinase, chloroplastic (Fragment) OS=Spinacia oleracea	45.572	+	-
19	P48497	Triosephosphate isomerase, cytosolic OS=Stellaria longipes	27.499	+	-
20	P93260	Glycolate oxidase OS=Mesembryanthemum crystallinum	40.612	+	-

21	Q9LEE0	Enolase OS=Spinacia oleracea	48.167	+	-
The above table continues					
22	Q9M3M0	NAD(P)H-quinone oxidoreductase subunit K, chloroplastic	25.557	+	-
23	T1D1B9	Hydroxypyruvate reductase (Fragment) OS=Silene latifolia	9.955	+	-
24	T1E1K3	Triosephosphate isomerase (Fragment) OS=Silene latifolia	16.187	+	-
25	A0A411L2V2	ATP synthase subunit alpha, chloroplastic OS=Froelichia latifolia	55.562	+	-
26	A0A0K9RQ86	ATP synthase subunit beta OS=Spinacia oleracea	59.387	+	-

Figure S1. The schematic gram of collecting secretion liquid of *L. bicolor* salt glands.

(a) Leaf discs were prepared using a 1 cm diameter puncher; (b) The abaxial surface of leaf discs was put on 200 mM NaCl solution; (c) Mineral oil was covered on the surface of the leaf discs with a rubber dropper; (d) Droplets formed on the leaf discs surface and collected by micropipette.

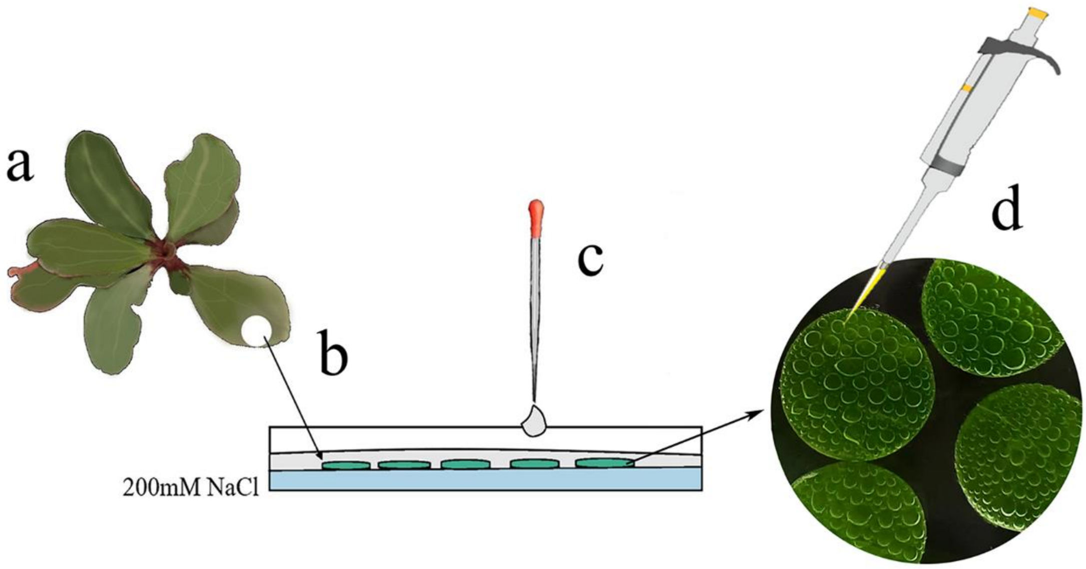

Figure S2. Evans blue staining of isolated leaf discs.

(a) The leaf discs without any treatment; (b) The leaf discs were treated by 200 mM NaCl solution 24h; (c) The leaf discs were achieved from the plant which treated by Hoagland's nutrient solution two weeks and the leaf discs were treated by 200 mM

NaCl solution 24h; (d) The leaf discs were achieved from the plant which treated by 200 mM NaCl solution two weeks and the leaf discs were treated by 200 mM NaCl Hoagland's nutrient solution 24h. Droplets formed on the leaf discs surface and collected by micropipette.

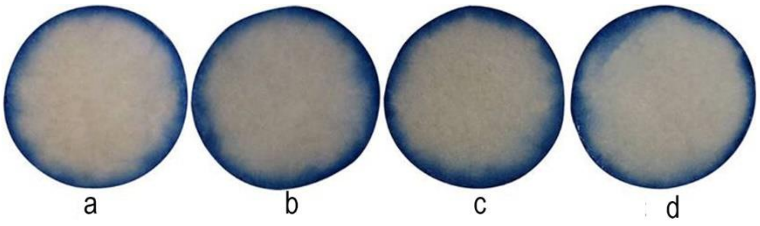

Figure S3. The secretion rate of Na^+, K^+ and Cl^- in single salt gland per hour.

Three replicates were used to calculate the standard deviation (SD) and ensure accuracy. Different letters (a–b) were used to indicate significant differences between different columns at $P < 0.05$ according to Duncan′s multiple range test.

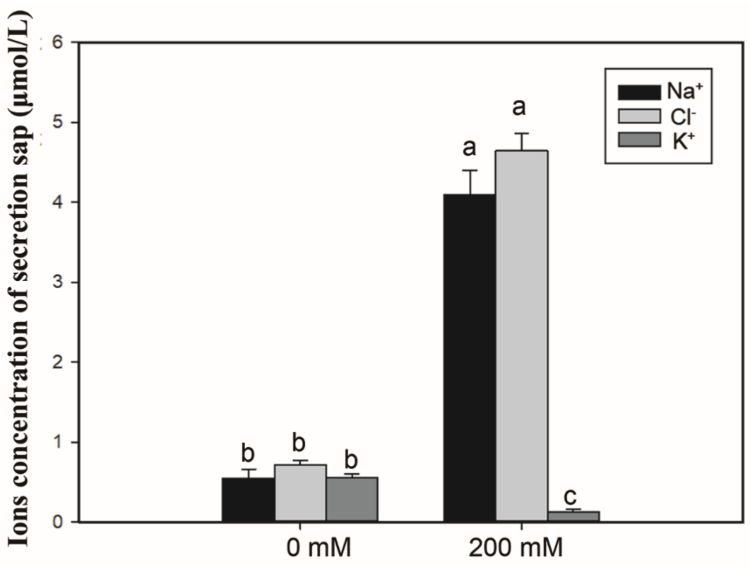

Figure S4. SDS-PAGE of proteins secreted by salt glands of *L. bicolor.*

M: Marker 10-180 kDa; Lane 1: control; Lane 2: 200 mM NaCl; Lane 3: control was diluted 5 times; Lane 4: control was diluted 10 times.

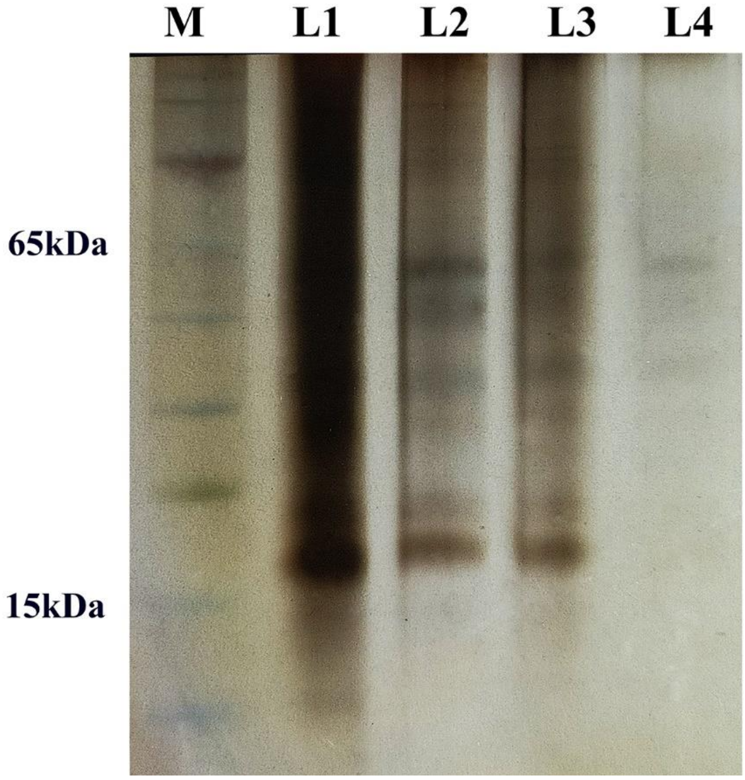
M
L1
L2
L3
L4
65kDa
15kDa
